# *Shigella* Infections in Household Contacts of Pediatric Shigellosis Patients in Rural Bangladesh

**DOI:** 10.3201/eid2111.150333

**Published:** 2015-11

**Authors:** Christine Marie George, Shahnawaz Ahmed, Kaisar A. Talukder, Ishrat J. Azmi, Jamie Perin, R. Bradley Sack, David A Sack, O. Colin Stine, Lauren Oldja, Mohammad Shahnaij, Subhra Chakraborty, Tahmina Parvin, Sazzadul Islam Bhuyian, Edward Bouwer, Xiaotong Zhang, Trisheeta N. Hasan, Sharmin J. Luna, Fatema Akter, Abu S.G. Faruque

**Affiliations:** Johns Hopkins University, Baltimore, Maryland, USA (C.M. George, J. Perin, R.B. Sack, D.A. Sack, L. Oldja, S. Chakraborty, E. Bouwer, X. Zhang);; icddb,b, Bangladesh (S. Ahmed, K.A. Talukder, I.J. Azmi, M. Shahnaij, T. Parvin, S.I. Bhuyian, T.N. Hasan, S.J. Luna, F. Akter, A.S.G. Faruque);; University of Maryland School of Medicine, Baltimore (O.C. Stine)

**Keywords:** *Shigella*, shigellosis, child, households, enteric infections, bacteria, Bangladesh, insect vectors, houseflies, antibacterial drugs, dysentery, water quality, hygiene, sanitation, vector-borne infections

## Abstract

To examine rates of *Shigella* infections in household contacts of pediatric shigellosis patients, we followed contacts and controls prospectively for 1 week after the index patient obtained care. Household contacts of patients were 44 times more likely to develop a *Shigella* infection than were control contacts (odds ratio 44.7, 95% CI 5.5–361.6); 29 (94%) household contacts of shigellosis patients were infected with the same species and serotype as the index patient’s. Pulsed-field gel electrophoresis showed that 14 (88%) of 16 with infected contacts had strains that were indistinguishable from or closely related to the index patient’s strain. Latrine area fly counts were higher in patient households compared with control households, and 2 patient household water samples were positive for *Shigella*. We show high susceptibility of household contacts of shigellosis patients to *Shigella* infections and found environmental risk factors to be targeted in future interventions.

In South Asia and Africa, an estimated 88.5 million diarrhea episodes are attributed to *Shigella* infections annually (*1*). Shigellosis occurs most often in children <5 years of age (*2*,*3*). Two recent multicountry studies found that Bangladesh has the highest rates of shigellosis (*4*,*5*). In the recent Global Enteric Multicenter Study (GEMS) conducted at a study site in Mirzapur, Bangladesh, *Shigella* was the third leading cause of moderate-to-severe diarrhea in children 12–23 months of age and the second leading cause of moderate-to-severe diarrhea in children 24–59 months of age (*5*). In addition, hospital-based surveillance of *Shigella* in Bangladesh found that patients from rural health facilities have higher rates of *Shigella* isolates than patients from urban facilities (e.g., 3% in urban Dhaka vs. 12% in rural Mirzapur during 2011) (*6*). Previous studies have identified risk factors for developing shigellosis, such as age (*7*–*9*), high fly counts (*10*–*12*), contaminated food (*13*,*14*), and recent overnight travel (*8*). Furthermore, a recent study in rural Bangladesh found that 10% of tube wells sampled had detectable *Shigella* (*15*).

Household studies have indicated that family members of shigellosis patients are at much higher risk for developing a *Shigella* infection than the general population (13–19 cases/100 shigellosis patient households vs. 7 cases/100 control households) (*7*,*8*). However, little research has been done to identify clinical and environmental transmission routes for *Shigella* infection in this susceptible population.

*Shigella* includes 4 species and numerous serotypes: *S. flexneri* (17 serotypes), *S. dysenteriae* (16 serotypes), *S. boydii* (20 serotypes), and *S. sonnei* (1 serotype) (*16*). A study in Wisconsin found that isolates from family members of index shigellosis patients were always the same serotype as the index patient’s (*7*). In contrast, studies in rural (*8*) and urban (*9*) Bangladesh found that 75% and 72%, respectively, of infected household contacts of shigellosis patients excreted serotypes different from the index patient’s serotype. These studies suggest that *Shigella* infections in Bangladesh are attributable to both secondary transmission and external infecting sources. To examine the rate of *Shigella* infection within households of shigellosis patients and to investigate risk factors for infection, we prospectively observed a cohort of household contacts of pediatric shigellosis patients and community controls in rural Mirzapur, Bangladesh.

## Methods

This study was conducted in Mirzapur, a subdistrict of Bangladesh’s Tangail district, at a field site of the icddr,b. Mirzapur is the Bangladesh site of the GEMS Demographic Surveillance System. We received ethical approval for this study from the icddr,b ethical review committee and an exemption from the Institutional Review Board at the Johns Hopkins Bloomberg School of Public Health (Baltimore, MD, USA). Written informed consent was obtained from all study participants or their guardians.

A cohort of household contacts of index shigellosis patients and matched community controls was followed prospectively for 7 days after each index patient visited the Kumudini Women’s Medical College and Hospital in Mirzapur for health care. Our sample size was determined by the number of pediatric shigellosis patients that could be recruited during October 2013–July 2014. Patients with suspected pediatric shigellosis were identified as children <5 years of age with dysentery, which was defined as having >1 stool containing blood during the previous 24 hours, as reported by a guardian or observed by research personnel. Community controls were matched to index shigellosis patients on the basis of age (within 3 months) and village location and were randomly selected by using the GEMS Demographic Surveillance System. Stool samples were collected from patients with suspected shigellosis and from community controls at time of enrollment in the study and were immediately sent in a cooler to the icddr,b Enteric and Food Microbiology Laboratory in Dhaka, Bangladesh, for bacterial culture analysis to detect *Shigella.*

Households of suspected shigellosis patients whose samples were found to be negative for *Shigella* by culture were excluded from the study. All index patients enrolled in the study received ciprofloxacin as part of their standard course of care at the Kumudini Hospital. 

After enrollment of pediatric shigellosis patients, we recruited household contacts of shigellosis patients and of community controls. Household contacts were defined as persons sharing the same cooking pot as the index shigellosis patient or control during the previous 3 days. To be eligible for the study, household contacts had to report that they would be present in the household of the patient or control for the next 7 days and be present for most household visits. Household contacts were followed prospectively for clinical and environmental surveillance by conducting household visits at days 1, 3, 5, and 7 after the initial visit of the index shigellosis patient Kumudini Hospital.

For clinical surveillance, household contacts were asked to report whether, during the previous 48 hours, they had diarrhea (>3 loose stools during a 24-hour period), dysentery (blood in stool observed by caregiver or research personnel), or vomiting. A stool sample was collected from enrolled household contacts at every visit. In addition, at each household visit, a questionnaire was administered to enrolled contacts to collect information on previously identified risk factors for *Shigella* infection. For environmental surveillance, on days 1 and 5, a water sample was collected directly from the household’s primary drinking water source, and a second sample was collected from drinking water stored in the home for immediate consumption. The water samples were tested for *Shigella* spp. by bacterial culture and PCR. Trained field assistants also conducted fly counts by using a Scudder grill at all surveillance visits over a period of 30 minutes at the kitchen and latrine area of each household, according to previously published methods (*12*). Weekly fly counts for each household were the total number of flies observed during surveillance visits.

All stool and water samples collected were analyzed at the icddr,b Enteric and Food Microbiology Laboratory in Dhaka. The laboratory received no information identifying whether samples were from patient or control households. For isolation of *Shigella*, stool and water samples were cultured on MacConkey and *Shigella-Salmonella* agar media, and *Shigella* was isolated and serotyped by using standard microbiologic and biochemical methods described previously (*4*). In brief, water samples of 1,000 mL were filtered through 0.22 μm pore-size filters. The filter paper was enriched in 25 mL gram-negative broth at 37°C overnight and then analyzed by culture. Template DNA was prepared from the enriched broth and tested for the *ipa*H gene, according to previously published methods (*16*).

To determine genetic relatedness of *Shigella* strains isolated within households, pulsed-field gel electrophoresis (PFGE) was performed on all *Shigella–*positive water and stool samples, according to the PulseNet protocol (*17*). Agarose*–*embedded genomic DNA of *Shigella* strains was digested by using *Xba*I, and fragments were separated by using a CHEF-DR II apparatus (Bio-Rad, Hercules, CA, USA) (*18*). Genetic relatedness was determined on the basis of previously published methods (*19*). To compare strains within a single household, 4 categories of genetic relatedness were used: a) “indistinguishable” (all fragments matched); b) “closely related” (1*–*3 fragments differed); c) “possibly related” (4*–*6 fragments differed); and d) “unrelated” (>7 fragments differed).

A *Shigella*-infected person was defined as a person with a stool sample positive for *Shigella* spp. by culture. Various tests were used for household-level variables: a McNemar test for paired binary variables; a Friedman test for clustered categorical variables with >2 levels; and a Wilcoxon signed-rank test for paired continuous variables (Tables 1, 2). For individual-level variables, a Fisher exact test was used for categorical variables and a Wilcoxon rank-sum test for continuous variables (Tables 1, 2). 

**Table 1:** Characteristics of household contacts of pediatric shigellosis patients and of community controls, rural Bangladesh

Characteristic	Median ± SD (range) or no. (%)		p value
	Patient contacts, n = 81	Control contacts, n = 77	
No. enrolled contacts per household*	3.0 ± 0.73 (2.0–5.0)	3.0 ± 0.95 (2.0–6.0)	0.25
No. persons living in the household for past 6 mo*	5.0 ± 1.4 (3.0–9.0)	6.0 ± 2.7 (3.0 –15.0)	0.16
Age of contacts, y†	27.0 ± 16.9 (1.8–72.0)	30.0 ± 18.6 (3.5–89.0)	0.47
Hours contacts spent outside their home in the past 48 h during surveillance period†	2.0 ± 1.8 (0–6.3)	1.0 ± 1.8 (0–7.3)	0.09
Female sex‡	48 (58)	46 (61)	0.75
Drank water outside their home during surveillance period‡	57 (69)	48 (62)	0.41
Consumed food outside their home during surveillance period‡	51 (61)	36 (47)	0.08
Consumed uncooked vegetables or fruits during surveillance period‡	22 (27)	12 (16)	0.12

*For household characteristics, a Wilcoxon signed-rank test was used for paired continuous variables. †For individual characteristics, a Wilcoxon rank-sum test was used for continuous variables.  ‡For individual characteristics, a Fisher exact test was used for categorical variables.

**Table 2:** Demographic and environmental characteristics of households of pediatric shigellosis patients and of community controls, rural Bangladesh

Characteristic	No. (%) or median ± SD (range)		p value
	Patient households, n = 27	Control households, n = 27	
Demographic*			
Age of child, patient or control†			
0–11 mo	3 (11)	3 (11)	1.00
12–23 mo	11 (41)	11 (41)	
24–35 mo	6 (22)	6 (22)	
36–47 mo	6 (22)	6 (22)	
48–59 mo	1 (4)	1 (4)	
Female sex, patient or control†	13 (48)	13 (48)	
Primary caregiver educational level‡			
No formal education	2 (7)	4 (15)	0.48
Less than primary school	2 (7)	3 (11)	
Completed primary school or greater	23 (86)	20 (74)	
Electricity in home*	20 (74)	19 (70)	0.75
Environmental			
Main source of drinking water*			
Shallow tube well	16 (59)	16 (59)	1.00
Deep tube well	11 (41)	11 (41)	
Households with water source S*higella* positive by PCR for *ipa*H gene*	0	2 (7)	0.48
Households with stored water S*higella* positive by culture*	2 (7)	0	0.48
Households with stored water S*higella* positive by PCR*	2 (7)	1 (4)	1.00
Households with no soap observed at any surveillance visit*§	18 (67)	19 (70)	0.75
Floor type*			
Earth	18 (67)	23 (85)	0.13
Concrete	9 (33)	4 (15)	
Latrine type‡			
Ventilated improved pit latrine	14 (52)	12 (44)	0.49
Pour flush toilet	6 (22)	6 (22)	
Traditional pit latrine	6 (22)	8 (30)	
No facility	1 (4)	1 (4)	
Latrine area weekly fly counts¶	27 ± 20 (0–84)	16 ± 13 (0–48)	0.0014
Kitchen area weekly fly counts¶	59 ± 55 (0–216)	44 ± 48 (0–192)	0.47

*McNemar test was used for paired categorical variables.  †All patient–control pairs were the same. ‡Friedman test was used for paired categorical variables with >2 levels.  §Soap within 10 steps of location reported to be used for household defecation.  ¶Wilcoxon signed-rank test was used for paired continuous variables.

Logistic regression was used to estimate the odds of developing a *Shigella* infection. Generalized estimating equations were used to account for clustering within households and in patient–control pairs and to estimate an odds ratio (OR) and approximate 95% CIs. Clusters in this analysis are the 27 patient–control pairs. A bivariate analysis was performed in which index patient or control child status in the household was used as the single predictor, and a binary outcome was used to determine whether household members developed a *Shigella* infection. All analyses were performed by using SAS, version 9.3 (SAS Institute, Inc., Cary, NC, USA).

## Results

During October 2013–July 2014, a total of 27 shigellosis patients with 83 household contacts and 27 community controls with 77 household contacts were followed prospectively. Of the initial 61 suspected shigellosis patients who were screened, 29 were excluded because cultures were negative for *Shigella*, and 5 were excluded because the caregiver was too busy or uninterested in the study. Of the 33 community controls screened, 6 were excluded because they did not pass stool on visit 1. Of 88 shigellosis–patient household contacts and 81 control household contacts who were screened for eligibility, 5 (6%) and 4 (5%), respectively, were excluded from the study because they did not pass stool on visit 1. 

Median age for household contacts was 27 years for patient households and 30 years for control households (Table 1). Of the 83 household contacts in patient households, 48 (58%) were women, compared with 47 (61%) of the 77 contacts in control households. Patient and control households did not differ significantly by age, sex, number enrolled, or number of total contacts (Table 1). Among household contacts of patients and control children, 52 (33%) were mothers, 31 (19%) fathers, 23 (14%) brothers, 22 (14%) sisters, and 32 (20%) other relatives. Contact relationship to the patient or control child did not differ significantly (p = 0.88). 

During the surveillance period, patient contacts reported spending more time outside the home than did control contacts (median time outside home during previous 48 hours, 2 hours vs. 1 hour; p = 0.09); eating more food outside the home (61% vs. 47%; p = 0.08); and consuming more uncooked vegetables and fruits (27% vs. 16%; p = 0.12). These differences were not statistically significant (Table 1). The rate for acquiring stool samples was 97% for enrolled household contacts and did not differ significantly for patient and control households (p = 0.15).

We found no significant differences between patient and control households in caregiver’s education level, type of latrine or floors, or presence of soap at the household latrine area, a proxy measure for handwashing with soap (Table 2). All households relied on tube wells as their primary drinking water source. The latrine areas of patient households had significantly higher weekly fly counts compared with those of control households (p = 0.001), but weekly kitchen fly counts did not differ significantly (p = 0.47). In patient households, 33% had concrete floors compared with 15% of control households; this difference was not statistically significant (p = 0.13). Household water samples from 2 (7%) patient households were positive by PCR for the *ipaH* gene of *Shigella* and were culture positive for non–type 1 *S. dysenterae* and *S. sonnei* during the surveillance period, compared with 1 PCR-positive (for the *ipaH* gene) household water sample and 2 PCR-positive samples from household water sources in control households.

Of the 27 patient households, 16 (59%) had >1 *Shigella*-infected contact during the 7-day surveillance period, compared with 1 (4%) control household, in which an asymptomatic *Shigella* infection was detected on day 1 of clinical surveillance (Table 3). In a bivariate model that used patient household as the predictor, the odds of developing a *Shigella* infection were 44 times higher for patient contacts than for control contacts (OR 44.7, 95% CI 5.5–361.6). The 16 patient households had 31 *Shigella-*infected contacts, compared with 1 *Shigella*-infected contact in 1 control household (Table 4). Four (15%) patient households had 6 contacts with symptomatic *Shigella* infection (i.e., having diarrhea, vomiting, or blood in stool during the previous 48 hours). *Shigella* infections for 13 (42%) of 31 *Shigella*-infected patient contacts (asymptomatic and symptomatic) were first detected on day 1 of clinical surveillance; 6 (19%) were detected on day 3, 5 (16%) on day 5, and 7 (23%) on day 7. 

**Table 3:** Household infection characteristics of pediatric shigellosis patients and of community controls, rural Bangladesh*

Characteristic	Patient households, no. (%), n = 27	Control households, no. (%), n = 27	p value†
Households with >1 infected contact	16 (59)	1 (4)	<0.0001
Households with >1 contact with infection at visit 1	9 (33)	1 (4)	0.02
Households with >1 contact with initial infection detected at visits other than visit 1	11 (41)	0	0.001
Households with >1 infected symptomatic contact‡	4 (15)	0	0.07
Households with >1 infected contact with same species and serotype as index patient’s	15 (94)	–	–
Households with >1 contact with different species and serotype than index patient’s	2 (12)	–	–

*–, not applicable because control households had no index patient.  †McNemar test was used for paired categorical variables, and Wilcoxon signed-rank test for continuous paired variables.  ‡Defined as a *Shigella* infection with diarrhea, vomiting, or blood in stool during previous 48 hours.

**Table 4:** Characteristics of household contacts with *Shigella* infections for pediatric shigellosis patients and community controls, rural Bangladesh

Characteristic	Patient contacts			Control contacts		p value*
	No. (%)	Total no.		No. (%)	Total no.	
Contacts infected	31 (37)	83		1 (1)	77	<0.0001
Contacts with symptomatic infections†	6 (7)	83		0	77	0.03
Contacts with infection detected on visit 1 of surveillance	13 (16)	83		1 (1)	77	0.0013
Contacts with infection detected on visits other than visit 1 of surveillance	18 (22)	83		0	77	
Infected contacts by sex						
M	18 (51)	35		0	31	0.44
F	13 (27)	48		1 (2)	46	
Infected contacts by relation to patient or control child						
Mother	9 (35)	26		0	26	0.09
Father	8 (53)	15		0	16	
Brother	6 (55	11		0	12	
Sister	2 (17)	12		1 (10)	10	
Other relative	6 (32)	19		0	13	
Infected contacts by *Shigella* species and serotype						
* S. flexneri*	20 (65)	31		0	1	0.63
*S. flexneri* 1b	2 (6)	31		0	1	
*S. flexneri* 1c	3 (10)	31		0	1	
*S. flexneri* 2a	12 (39)	31		0	1	
*S. flexneri* 3a	3 (10)	31		0	1	
*S. flexneri* 4X	0	31		0	1	
* S. sonnei*	9 (29)	31		1 (100)	1	
* S. boydii*	2 (6)	31		0	0	
*S. boydii* 7	1 (3)	31		0	0	
* S. boydii *	1 (3)	31		0	0	
* S. dysenteriae*	0	31		0	0	

*By Fisher exact test.  †Defined as a *Shigella* infection with diarrhea, vomiting, or blood in stool during previous 48 hours.

In patient households, a *Shigella* infection developed in 18 (51%) of 35 male contacts and in 13 (27%) of 48 female contacts during the surveillance period (p = 0.02). Five of the 6 symptomatic *Shigella* infections were in men, and 4 of the infections were first detected on day 1 or 3 of surveillance. Difference in day of initial detection by sex was not significant (p = 0.53), but male contacts spent significantly more time outside their homes during the surveillance period (p<0.0001) and reported drinking significantly more water outside their homes (p<0.0001) than did female contacts.

During the surveillance period, 4 patient contacts reported using antibacterial drugs; 3 of those had a symptomatic *Shigella* infection, 1 of whom was hospitalized for symptoms. This person tested positive on visit 1. To estimate duration of shedding for patient household contacts, we observed the time during which shedding occurred for 6 household contacts with a *Shigella* infection first detected on visit 2. Of these 6 contacts, 5 (83%) had detectable shedding for <2 days: 3 for 1 day, 2 for 2 days, and 1 for 3 days. 

Among the 31 patient household contacts in whom *Shigella* infection developed, 29 (94%) were infected with the same species and serotype as the index patient in their household (Table 5). Twenty (65%) of the 31 patient household contacts with detectable *Shigella* in stool by culture had *S. flexneri* (2 *S. flexneri* 1b, 3 *S. flexneri* 1c, 12 *S. flexneri* 2a, and 3 *S.flexneri* 3a); 9 (29%) had *S*. *sonnei*; and 2 (6%) had *S. bodyii* (1 *S. boydii* 7 and 1 *S. boydii* of an unknown serotype) (*16**–**18*). In the 16 patient households with infected household contacts, 7 (44%) contacts had strains indistinguishable from those of the index patient by PFGE analysis; 7 (44%) had closely related strains; and 2 (12%) had unrelated strains (Table 5).

**Table 5:** Patient households with *Shigella*-infected household contacts by *Shigella* species and serotype and visit number at which infection was detected, rural Bangladesh*

Household	Household member	Species and serotype	Visit no.	No. PFGE genotypes within household	Household genetic relatedness of strains†
Household 1	Patient	*S. flexneri *2a	1	2	Closely related
	Contact 1	*S. flexneri *2a	3		
	Contact 2	*S. flexneri *2a	1		
	Contact 2	*S. flexneri *2a	2		
Household 2	Patient	*S. flexneri *2a	1	2	Unrelated
	Contact 1	*S. flexneri *2a	3		
	Contact 1	*S. flexneri *2a	4		
	Contact 2	*S. boydii *	1		
Household 3	Patient	*S. sonnei*	1	1	Indistinguishable
	Contact 1	*S. sonnei*	1		
	Contact 1	*S. sonnei*	2		
	Contact 2	*S. sonnei*	4		
	Stored water	*S. sonnei*	1		
Household 4	Patient	*S. sonnei*	1	1	Indistinguishable
	Contact 1	*S. sonnei*	1		
	Contact 1	*S. sonnei*	2		
Household 5	Patient	*S. flexneri *2a	1	2	Closely related
	Contact 1	*S. flexneri *2a	4		
	Contact 2	*S. flexneri *2a	2		
	Contact 2	*S. flexneri *2a	3		
	Contact 2	*S. flexneri *2a	4		
Household 6	Patient	*S. flexneri *2a	1	2	Closely related
	Contact 1	*S. flexneri *2a	1		
	Contact 1	*S. flexneri *2a	2		
	Contact 2	*S. flexneri *2a	1		
	Contact 2	*S. flexneri *2a	2		
	Contact 3	*S. flexneri *2a	1		
Household 7	Patient	*S. flexneri *1c	1	3	Closely related
	Contact 1	*S. flexneri *1c	4		
	Contact 2	*S. flexneri *1c	3		
	Contact 3	*S. flexneri *1c	2		
	Contact 3	*S. flexneri *1c	4		
Household 8	Patient	*S. flexneri *4X	1	2	Unrelated
	Contact 1	*S. boydii *7	2		
Household 9	Patient	*S. sonnei*	1	1	Indistinguishable
	Contact 1	*S. sonnei*	1		
	Contact 1	*S. sonnei*	2		
	Contact 2	*S. sonnei*	2		
Household 10	Patient	*S. flexneri *1b	1	3	Closely related
	Contact 1	*S. flexneri *1b	1		
	Contact 1	*S. flexneri *1b	2		
	Contact 1	*S. flexneri *1b	3		
	Contact 2	*S. flexneri *1b	1		
Household 11	Patient	*S. flexneri *2a	1	1	Indistinguishable
	Contact 1	*S. flexneri *2a	3		
	Contact 1	*S. flexneri *2a	4		
	Contact 2	*S. flexneri *2a	3		
Household 12	Patient	*S. flexneri *3a	1	1	Indistinguishable
	Contact 1	*S. flexneri *3a	4		
	Contact 2	*S. flexneri *3a	4		
	Contact 3	*S. flexneri *3a	2		
Household 13	Patient	*S. sonnei*	1	2	Closely related
	Contact 1	*S. sonnei*	2		
	Contact 1	*S. sonnei*	3		
Household 14	Patient	*S. sonnei*	1	1	Indistinguishable
	Contact 1	*S. sonnei*	1		
	Contact 1	*S. sonnei*	2		
Household 15	Patient	*S. flexneri *2a	1	2	Closely related
	Contact 1	*S. flexneri *2a	1		
	Contact 2	*S. flexneri *2a	1		
Household 16	Patient	*S. sonnei*	1	1	Indistinguishable
	Contact 1	*S. sonnei*	4		
	Contact 2	*S. sonnei*	4		
*Excluding household contact with different *Shigella* species. PFGE, pulsed-field gel electrophoresis. †Strain relatedness determined by using criteria in (*19*).					

## Discussion

We found that the odds of developing a *Shigella* infection were >44 times higher for contacts of pediatric shigellosis patients than for control contacts (OR 44.7, 95% CI 5.5–361.6). We also observed that 94% of patient household contacts had the same species and serotype as the index patient. Consistent with this finding, PFGE analysis found that 88% of households with infected household contacts had strains that were indistinguishable from or closely related to the index patient’s strain. In contrast, 2 previous studies in Bangladesh found that only one quarter of household contacts had the same species and serotype as the index patient in their home (*8*,*9*). Our finding suggests that a single infectious pathogen is being spread in study households; however, whether the infections are caused by a shared environmental source or secondary transmission from an infected household member is unclear.

We observed that most (59%) patient households had >1 household contact with a *Shigella* infection over the 7-day surveillance period, and 25% of these households had contacts who had symptomatic infections. In comparison, 1 (4%) control household contact had a *Shigella* infection, which was asymptomatic. The proportion of shigellosis patient households with *Shigella*-infected contacts in our study is higher than has been previously reported. In a study in Peru, 34% of households of index shigellosis patients during the 1-week surveillance period had infected contacts (*20*). Another study conducted in rural Bangladesh in 1974 found that 19% of members of household compounds (i.e., multiple households living together) developed *Shigella* infection over a 10-day period after identification of the index shigellosis patient, compared with 7% of members of control household compounds (*21*). The reason our study found a higher rate of infection in household contacts of shigellosis patients is unknown but may reflect differences in environmental risk factors or population immunity.

Despite no significant difference in toilet type, we found significantly higher fly counts in the latrine areas of patient households than in control households during the surveillance period (Table 2). This finding likely suggests that patient households had latrines with poorer sanitary conditions than did control households. Similarly, a recent study conducted in the same site found a significant association between seasonal fly densities and peaks in pediatric shigellosis patients (*12*). Furthermore, these significant associations are consistent with the growing body of literature that implicates houseflies as vectors of shigellosis. The 2 flies thought to be responsible for transmission of *Shigella* are *Musca domestica*, because of its mobility, and *M. sorbens*, because it commonly breeds in human feces. Previous studies in Bangladesh, Myanmar, Egypt, and the United States have detected *Shigella* in flies by culture (*11*). An intervention study conducted on 2 military field bases in Israel found that baited fly traps reduced fly counts by 64% and rates of shigellosis by 85% compared with fly counts and shigellosis rates on the control base (*10*). These studies suggest that fly control could be a promising intervention to reduce *Shigella* transmission in the study population. Future studies should more closely evaluate the potential of flies to be a vector of *Shigella* in our study population by culturing flies and conducting fly species identification.

Our study also found 2 (7%) patient households with stored water samples positive for *Shigella*. In 1 household, detectable *S. sonnei* by culture was found in stool from the patient and 2 household contacts and in stored water. This finding suggests potential secondary contamination of stored water by a household member, particularly because none of the corresponding source water samples had detectable *Shigella* by culture. In another household, *S. dysenteriae* was found in stored water but was not detected in any household members. A potential explanation for this finding is that the *Shigella* came from the household tube well. A previous study in rural Bangladesh identified *Shigella* by PCR in 10% of tube wells; that study also found that 40% of these tube wells were contaminated with rotavirus, 10% with *Vibrio* spp., and 8% with pathogenic *Escherichia coli* (*15*). The mostly likely source of *Shigella* in tube well water is fecal contamination from latrines, which are commonly located near tube wells used for drinking in rural Bangladesh. Further research is needed to evaluate whether groundwater is a major environmental transmission route for shigellosis and other enteric infections in this population.

We observed that male patient contacts were twice as likely as female contacts to develop *Shigella* infection during the surveillance period (51% vs. 27%); all but 1 symptomatic infections in contacts were in men. The reason for this higher rate of infection among male household contacts is unknown, but a possible explanation is that men may introduce the infection into the home. Future studies should investigate the role of sex in susceptibility and transmission of *Shigella* infection.

Among patient households, 41% had >1 contact who developed an initial *Shigella* infection after day 1 of surveillance. This finding suggests a potential opportunity to intervene in *Shigella* transmission in households with shigellosis patients. An intervention study that promoted handwashing with soap in Dhaka reduced the secondary infection rate for *Shigella* by 69% in the 10-day period after identification of the index patient compared with a control group (*22*). Future studies should evaluate whether this intervention would be effective in rural settings such as Mirzapur.

This study has several limitations. First, our small sample size limited our ability to detect significant differences in environmental risk factors for *Shigella* infection at the household level and to detect differences in behavioral risk factors at the individual level. Second, our analysis focused on pediatric index shigellosis patients, so our findings are not necessarily generalizable to older index patients. Third, we did not collect longitudinal stool samples from index patients and therefore cannot determine how long their shedding may have continued through the 1-week surveillance period. However, because all index patients received antibacterial drugs, we suspect that the shedding was minimal and that the source of *Shigella* infection in these households during the surveillance period was more likely from household members already infected or from a shared environmental source that we did not measure. Fourth, we used bacterial culture to detect *Shigella* in the stool samples. This method limited our analysis to infections with sufficiently high bacteria quantity in stool to be detected by culture. Future studies should use bacterial culture and quantitative PCR on collected stool samples. Finally, future studies should obtain >1 isolate from stool samples collected from each household contact to determine whether a person can shed multiple PFGE genotypes.

A main strength of our study was the environmental surveillance of household water sources, stored household water, and fly counts in study households. Second, we included households of both shigellosis patients and controls; this approach enabled us to examine rates of *Shigella* infection in patient households, compared with control households, and to investigate household-level risk factors for shigellosis. Third, we followed up with study households at 4 specific times during a 1-week period to obtain detailed information on potential behavioral and environmental risk factors for *Shigella* infection. Fourth, we used PFGE to conduct genetic characterization of strains within households.

In rural Bangladesh, household contacts of shigellosis patients are highly susceptible to *Shigella* infection during the week after the index patient visits a health facility for care. Our findings suggest that each shigellosis patient household represents the spread of a single infectious pathogen. Therefore, interventions for household-level risk factors, such as fly control, water treatment, and hygiene practices, could potentially reduce *Shigella* transmission in this high-risk population.
